# A Liposome-Based Approach to the Integrated Multi-Component Antigen Microarrays

**DOI:** 10.3390/microarrays4040618

**Published:** 2015-11-20

**Authors:** Denong Wang

**Affiliations:** Tumor Glycomics Laboratory, SRI International Biosciences Division, Menlo Park, CA 94025, USA; E-Mail: denong.wang@sri.com; Tel.: +1-650-859-2789; Fax: +1-650-859-3153

**Keywords:** lipids, liposome, liposome microarrays, antigen microarrays, natural antibodies, autoantibodies, high-throughput serology, encephalomyelitis

## Abstract

This report describes an experimental procedure for constructing integrated lipid, carbohydrate, and protein microarrays. In essence, it prints liposomes on nitrocellulose-coated micro-glass slides, a biochip substrate for spotting protein and carbohydrate microarrays, and the substances that can form liposomes (*homo*-liposomes) or can be incorporated into liposomes (*hetero*-liposomes) are suitable for microarray construction using existing microarray spotting devices. Importantly, this technology allows simultaneous detection of serum antibody activities among the three major classes of antigens, *i.e.*, lipids, carbohydrates, and proteins. The potential of this technology is illustrated by its use in revealing a broad-spectrum of pre-existing anti-lipid antibodies in blood circulation and monitoring the epitope spreading of autoantibody reactivities among protein, carbohydrate, and lipid antigens in experimental autoimmune encephalomyelitis (EAE).

## 1. Introduction

Like proteins and carbohydrates, lipids are a category of the essential elements of living cells. Lipid molecules of diverse structures are also important targets for immunological recognition and antibody responses [1,2,3]. Many bacterial pathogens produce phospholipids, glycolipids and/or lipopolysaccharides (LPS) of distinct antigenic structures [3,4]. Some are specific for a given pathogen and thereby serve as immunological targets for pathogen identification and diagnosis of infectious diseases, and as vaccines for the induction of anti-infection immune responses. There are also conserved lipid moieties among microbes, such as lipid A components of LPS, which are ligands of the Toll-like receptors of the innate immune system [5,6]. Host recognition of such lipids leads to rapid first-line anti-infection responses.

Lipid moieties of cellular components may also be molecular targets of autoimmune diseases [7,8,9,10,11,12,13]. In systemic lupus erythematosus (SLE), anti-cardiolipin antibodies have been detected in addition to the autoantibodies to protein and nucleic acid components [9]. In multiple sclerosis (MS) and autoimmune encephalomyelitis (EAE), there are increased T cell and autoantibody reactivities that are directed at myelin lipids [10,12,14]. A major histocompatibility (MHC)-like protein, CD1, binds certain types of lipid molecules and presents them to T cells or NK cells [2]. CD1 expression is increased at the site of brain lesions in both MS and its rodent model, EAE [10,11]. These autoimmune responses may be responsible for demyelination in central and/or peripheral neural tissues [12,13].

Lipid-based antigenic cross-reactivities or molecular mimicry between cellular components and specific microbial antigens may contribute to either pathogenesis of infectious diseases or clearance of cellular lipid products [7,8,15,16,17]. *Campylobacter jejuni* infection induced an autoimmune neurological disorder, Guillain-Barré syndrome, in about a third of cases [7,8]. This pathogen expresses a lipopolysaccharide molecule that mimics various gangliosides present in high concentrations in peripheral nerves. Infection by this bacterium may, thus, elicit undesired autoimmune responses to gangliosides of the host tissue. In addition, numerous viral infections induce this syndrome, since viruses collect gangliosides as they incorporate plasma membrane from the host cell.

Moreover, self-lipid components may be modified to generate neo-immunogenic lipid epitopes [16,17,18,19]. For example, oxidation of low-density lipoprotein (LDL) generates a variety of oxidatively modified lipids and lipid-protein adducts that are immunogenic and proinflammatory, which in turn contribute to atherogenesis [16,17]. Cells undergoing apoptosis also display oxidized moieties on their surface membranes, as determined by binding of oxidation-specific monoclonal antibodies. Some anti-lipid autoantibodies play roles in the clearance of non-essential or harmful cellular lipid derivatives and are, in fact, beneficial to the hosts [16,19,20]. Immunization with cell-wall polysaccharide of *Streptococcus pneumoniae* elicited T15 anti-phosphorylcholine antibodies, which cross-react with oxidized epitopes of low-density lipoprotein (oxLDL). Interestingly, this antibody response was found to be effective in eliminating oxLDL in circulation and in atherosclerotic lesions [17,19].

In summary, lipids represent an important class of biomolecules that are structurally diverse and of immunological significance. There is an increasing need to integrate the lipid component into antigen microarray platforms to facilitate characterization of lipid antigens and anti-lipid antibody responses. A number of researchers, including our team, have been using a highly efficient method for constructing protein and carbohydrate microarrays, *i.e.*, spotting carbohydrate and protein molecules on the nitrocellulose-coated micro-glass slide [21,22,23]. In this study, a practical experimental procedure was explored to enable high-throughput production of lipid microarrays using this biochip substrate.

## 2. Experimental Section

A technical barrier to production of lipid microarrays using nitrocellulose substrate is that most lipid molecules need to be solubilized in organic solvents. However, the existing micro-spotting devices were not designed to handle organic solvents. An experimental approach to overcome this problem is to spot the aqueous suspension of lipid vesicles, liposomes, on nitrocellulose-coated micro-glass slides [24]. Our general experimental procedures for liposome production and construction of the integrated multi-component antigen microarrays are outlined here.

### 2.1. Preparation of Liposomes

A probe-sonication protocol was applied with minor modifications to produce small, unilamellar vesicles [24,25]. In brief, the lipid solutions were dried down by method of nitrogen evaporation and then re-suspended in saline by vigorously vortexing to produce a milky, uniform lipid suspension that was sonicated on ice with a probe sonicator (VirSonic 475 of VIRTIS, Durham, NC USA) to produce transparent liposome preparations suited for liposome array construction.

Two types of liposomes, *homo-* and *hetero-*liposomes, were produced using this procedure. The former were made via a single lipid preparation, e.g., phosphatidylcholine (PTC), cerebroside, and sulfatide. The latter contained two different lipid molecules with PTC as the support to display other lipid/glycolipid in desired ratios or epitope densities. For example, a *hetero-*liposome of sulfatide (Supplementary Table S1, Antigen Index #20) was prepared with sulfatide and PTC at a ratio of 1:10 (*w/w*), *i.e.*, 0.2 mg sulfatide and 2.0 mg PTC per ml of liposome suspension in saline. Briefly, this liposome was named as Sulfatide/PTC_1/10. Compositions of all liposome preparations are given in Supplementary Table S1.

### 2.2. Printing Protein, Carbohydrate, and Lipid/Liposome Microarrays

A high-precision robot designed to produce cDNA microarrays (GMS 417 Arrayer; Genetic Microsystems, Inc., Woburn, MA, USA) was utilized to spot antigen preparations, including proteins/peptides, carbohydrates, and liposomes of various compositions onto the glass slides pre-coated with nitrocellulose polymer (FAST Slides; Schleicher & Schuell, Keene, NH, USA). Proteins and carbohydrates were dissolved in PBS (pH 7.4) and saline (0.9% NaCl), respectively. Liposome preparations are generally suspended in saline at concentrations as specified in Table S1. They were printed with spot sizes of ~150 μm and at 375-μm intervals, center to center. The printed microarrays were air-dried and stored at room temperature before application.

### 2.3. Microarray Assays

Immediately before use, the printed microarrays were rinsed with PBS, pH 7.4, with 0.05% (*v/v*) Tween 20 and then blocked by incubating the slides in 1% (*w/v*) BSA in PBS containing 0.05% (*w/v*) NaN_3_ for 30 min. They were then incubated with antibodies diluted in 1% (*w/v*) BSA in PBS containing 0.05% (*w/v*) NaN3 and 0.05% (*v/v*) Tween 20. Each array was first stained with a serum sample at a 1:25 dilution from a mouse with EAE or age-matched control SJ/L mouse. The captured IgG was stained with an anti-IgG antibody conjugated with Cy5 at 2 μg/mL and the captured IgM in the same array was revealed by an R-PE-tagged anti-IgM secondary antibody at 2 μg/mL (Rockland Immunochemicals, Inc., Pottstown, PA, USA). The stained slides were rinsed five times with PBS with 0.05% (*v/v*) Tween 20, air-dried at room temperature, and then scanned for fluorescent signals. The stained microarrays were scanned with ScanArray5000A Microarray Scanner (PerkinElmer Life Science, Boston, MA, USA) following the procedure in the manufacturer’s user manual. SAS Institute’s JMP-Genomics 6.0 (Cary, NC, USA) was applied for further statistical analysis as described in the figure legends.

## 3. Results and Discussion

A key question for this liposome array technology is whether the spotted liposomes preserve the antigenic determinants that are readily reactive with specific anti-lipid antibodies. It is noteworthy that anti-lipid antibodies are generally present in the repertoire of the murine natural antibody [26,27,28] and that a spectrum of autoantibodies targeting multiple classes of antigens was identified in an EAE model [12,14,29]. Thus, if the liposome arrays produced by this procedure preserve the lipid epitopes that are readily reactive with anti-lipid antibodies and if the assay reaches the sensitivity to detect these antibodies in blood circulation, using these arrays to scan the serum specimens collected from either normal mice or EAE subjects would allow detection of corresponding anti-lipid antibodies in these models. As summarized below, these assumptions were tested experimentally.

### 3.1. Homo-Liposome Arrays

**Figure 1:**
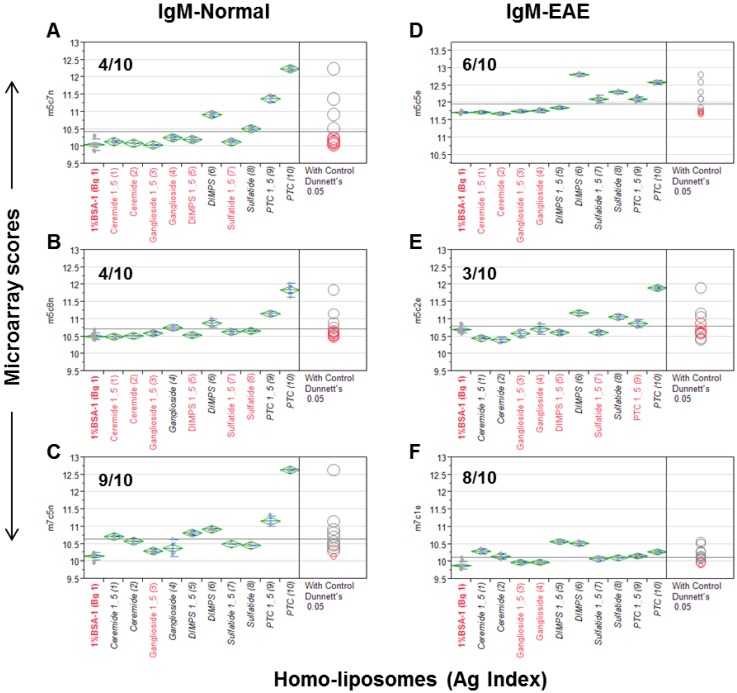
The *homo-*liposome microarray detected significant levels of anti-lipid antibodies in the natural antibody repertoire of SJL/J (**A**–**C**) and an EAE (experimental autoimmune encephalomyelitis) model (PLP-SJL/J) (**D**–**F**). Each array was stained with a serum sample at a 1:25 dilution from a mouse with EAE (**right**) or an age-matched control SJ/L mouse (**left**). The captured IgM was revealed by an R-PE-tagged anti-IgM secondary antibody at 2 μg/mL. Results are presented as microarray scores and statistically analyzed using SAS Institute’s JMP Genomics software package. Dunnett’s test was performed to determine whether the means of the triplicate liposome array detection were significantly different from the means of the background values (Bg. 1, *n* = 12). The means of the multiple points are shown as horizontal green bars. The top and bottom of the green diamonds represent the limits of the 95% confidence intervals for the means. The comparison circles for the results of Dunnett’s test appear to the right of the mean diamonds to illustrate the significance of the differences among the means. These circles allow visual inspection of the statistical significance of the differences. The more the circles intersect, the less significant their difference and vice versa. The color of the comparison circles and the color of the liposome name listed in *x*-axis are identified in black to indicate that the means of detection is significantly different from those of the means of Bg. 1 and red to refer to non-significant detection as compared to the background. The anti-lipid profiles appear to be different among the six subjects.

In the first step, we examined “*homo*-liposomes,” which are composed using a homogeneous lipid preparation. Five *homo*-liposome preparations—cerebroside, ganglioside, dimyristoylphosphatidylserine (DMPS), sulfatide, and PTC—were applied to the nitrocellulose slides at two dilutions producing 10 arrays of *homo*-liposomes for antibody staining (Figure 1). Since a number of anti-lipid antibodies, such as anti-PTC and anti-sulfatide, are often present in murine serum antibody repertoires, we probed the liposome arrays using a panel of mouse sera. These include three sera obtained from an EAE model and three from the age-matched SJL/J mice.

Figure 1 illustrates a quantitative analysis of each microarray assay. The results are presented as a group of six scatterplots, which were obtained by an antigen-by-antigen one-way analysis of variance (ANOVA). The microarray scores in the y-axis are the log2-transformed mean fluorescent intensities (MFIs); antigens and a background control (Bg. 1) are listed in *x*-axis. Dunnett’s test was performed to determine whether the means of a given liposome detection were significantly different from the means of Bg. 1. The levels of significance of detection are visibly illustrated in two ways, *i.e.*, the color and location of comparison circles and the color of the item name listed in x-axis, either in black (statistically significant) or red (not statistically significant). Since each circle was centered on the mean value for a specific antigen preparation and has a diameter that is proportional to standard error for the mean, the distance between an antigen’s circle and that of Bg. 1 quantitatively reflect the level of significance of the antibody signal detected by the antigen. The more the circles intersect, the less significant their difference, and vice versa. The horizontal solid line in the plot is the mean of the antibody responses for each array.

The numbers of positive detections that are statistically significant as compared to the background are shown in each plot. In Figure 1A–C, 4/10, 4/10, and 9/10 liposomes were positively stained by the IgM antibodies obtained from three normal SJL/J mice. Figure 1D–F show that 6/10, 3/10, and 8/10 liposomes were positively stained by three samples of EAE-derived IgM anti-sera. Anti-PTC, anti-sulfatide, and anti-DMPS antibodies were positive in all six mice; anti-ceramide antibodies were positive in one SJL/J and one EAE mouse; and anti-gangliosides were detected in two of the three normal SJL/J mice but not in EAE mice.

This antisera scanning experiment demonstrates that all the five *homo*-liposome preparations display the antigenic determinants on chip that are readily reactive with serum IgM antibodies of SJL/L mice, either from normal mice or the EAE model or from both normal controls and EAE. In striking contrast to these IgM-anti-lipid antibody profiles, anti-lipid IgG antibody was not detected in these samples.

### 3.2. Hetero-Liposome Arrays

Unlike *homo-*liposomes, which are produced by a single lipid preparation, a *hetero-*liposome contains at least two different lipid molecules. The configuration of *hetero-*liposomes resembles certain feature of cell membrane decorated with the polar moieties of lipids, such as phospholipids and complex sugar chains. We examined whether PTC liposomes can serve as a vesicle to carry other lipid molecules for spotting to extend the spectrum of antigenic determinants displayed by liposome arrays. The set of antisera applied in Figure 1 was used to examine whether a *hetero*-liposome may capture significant levels of anti-lipid antibodies. In this analysis, the microarray scores of PTC-liposome served as background values in the ANOVA to determine the antibody activities associated with corresponding *hetero*-liposome preparations that differ from anti-PTC antibody activity.

**Figure 2:**
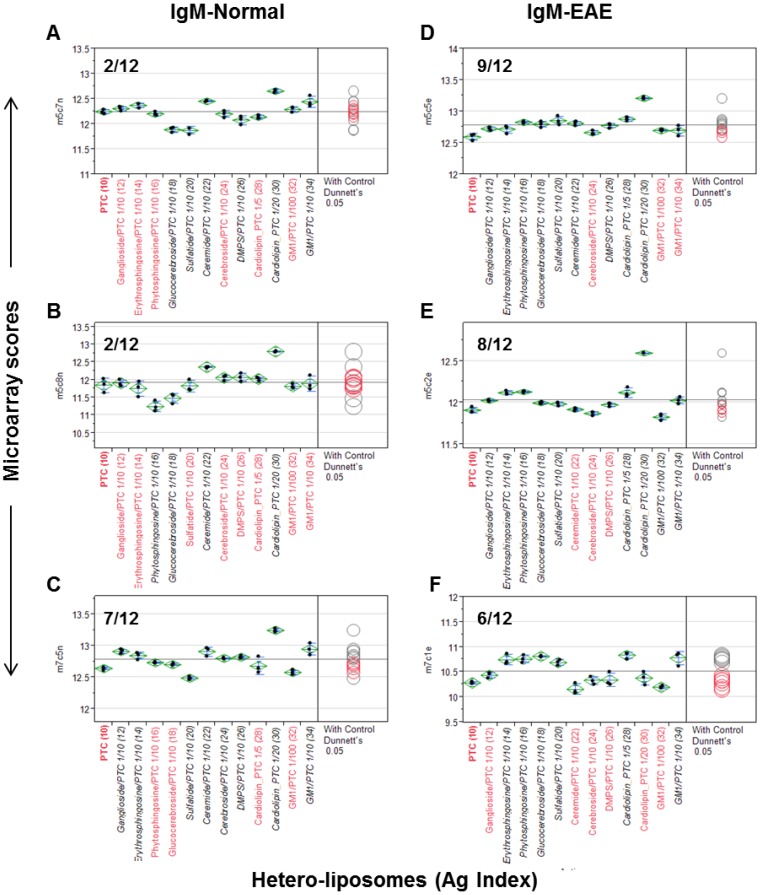
*Hetero-*liposome microarray captures the spectrum of anti-lipid antibodies in an EAE model (**D**–**F**) and SJL/J controls (**A**–**C**). Microarray staining and data analysis were performed as described in the Figure 1 legend. Dunnett’s test was performed to determine whether the means of given *hetero*-liposome detection was significantly different from the means of IgM signal captured by the PTC-stock *homo*-liposome.

As shown in Figure 2, the three normal control antisera detected 2/12, 2/12, and 7/12 positives and the three EAE antisera captured 9/12, 8/12, and 6/12 positives, respectively. Among lipid antigens, ganglioside/PTC was positive in 3/6 cases, ceramide/PTC in 4/6, cerebroside/PTC in 1/6, cardiolipin/PTC_1/20 in 5/6, GM1/PTC_1/100 in 0/6, and GM1/PTC_1/10 in 4/6 cases. glucocerebrosides/PTC, salfatide/PTC, cardiolipin/PTC, erythrosphingosine/PTC, and phytosphingosine/PTC were positive in all EAE but negative in the controls. Thus, 11/12 *hetero*-liposomes captured significant levels of anti-lipid IgM antibodies in at least one of the anti-sera. Since GM1/PTC 1/100 differs from GM1/PTC 1/10 only in the amounts of GM1 incorporated, the negative result of the former may reflect that its concentration or epitope density is too low to detect serum anti-GM1 antibodies.

Although both types of liposomes captured serum anti-lipid antibodies, the frequency of positives captured by *hetero*-liposomes appeared higher in the EAE group than those detected in the control group. Nevertheless, this observation needs to be validated in a larger sample size in order to determine whether any of these *hetero*-liposomes display valuable biomarkers for monitoring autoimmune disorders.

### 3.3. Capturing Personalized Antibody Profiles Using Integrated Multi-Component Microarrays

One of the important applications of this versatile biochip platform is for large-scale antibody profiling. This potential was investigated by constructing a set of customized lipid, carbohydrate, and protein microarrays and applying these arrays to explore the serum antibody repertoire.

**Figure 3:**
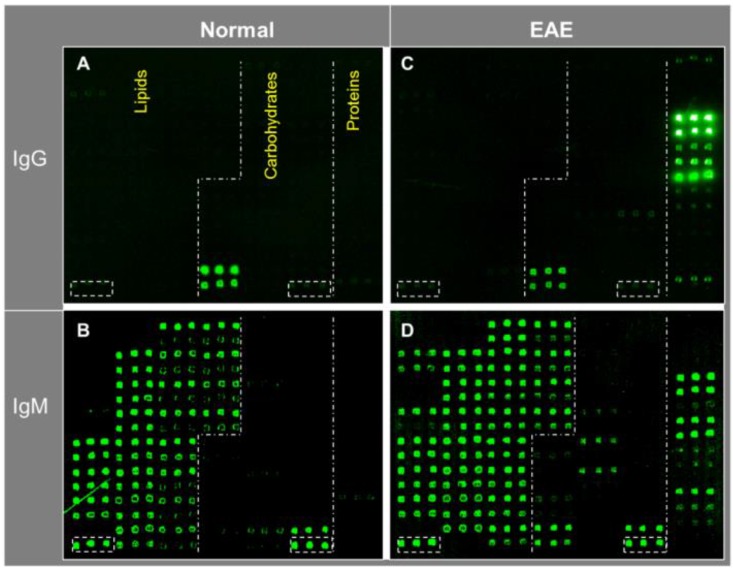
A versatile bioarray platform displays a diverse panel of proteins, peptides, carbohydrates, and lipids/liposomes for monitoring natural antibodies (**A**,**B**); and the autoimmune responses in an EAE animal model (**C**,**D**). As outlined by dashed lines in the array images, antigens were spotted in three zones, *i.e.*, lipids, carbohydrates, and proteins. Serum antibodies collected from previous EAE studies were analyzed. The EAE model was induced by challenging SJL/J mice with myelin proteolipid protein (PLP), amino acid residue 139–150, emulsified in complete Freund’s adjuvant (CFA) [14]. The antigen-specific antibody signal was revealed by co-staining the arrays with fluorophore-tagged anti-murine IgG (Cy5) and anti-IgM (R-PE) secondary antibodies as specified in the methods. The boxed spots in each array were a preparation of spotting positive control (P1). (**upper panel**: IgG; **bottom panel***:* IgM).

Figure 3 shows an example of such multi-component antigen arrays of 104 features, including 52 preparations of lipids/liposomes, 39 carbohydrates, and 13 proteins. Each class of antigens was spotted in the corresponding array zone as illustrated in the figure. Visual inspection of these microarray images readily reveals arrays of triplicate positive spots in each antigen zone. It is striking that the natural antibody repertoire in the non-immunized SJL/J mouse is predominately anti-lipid IgM antibodies (Figure 3B); only six spots of strong positives were detected in IgG panel—they were anti-Levan antibodies in the carbohydrate zone (Figure 3A). However, arrays of additional positives appeared in all the three antigen zones in the EAE-IgM panel (Figure 3D). Moreover, multiple arrays of strong positive IgG spots were captured in the protein zone in the EAE sample (Figure 3C). Thus, this technology is sensitive enough to specifically detect anti-lipid, anti-carbohydrate, and anti-protein antibodies in blood circulation.

**Figure 4:**
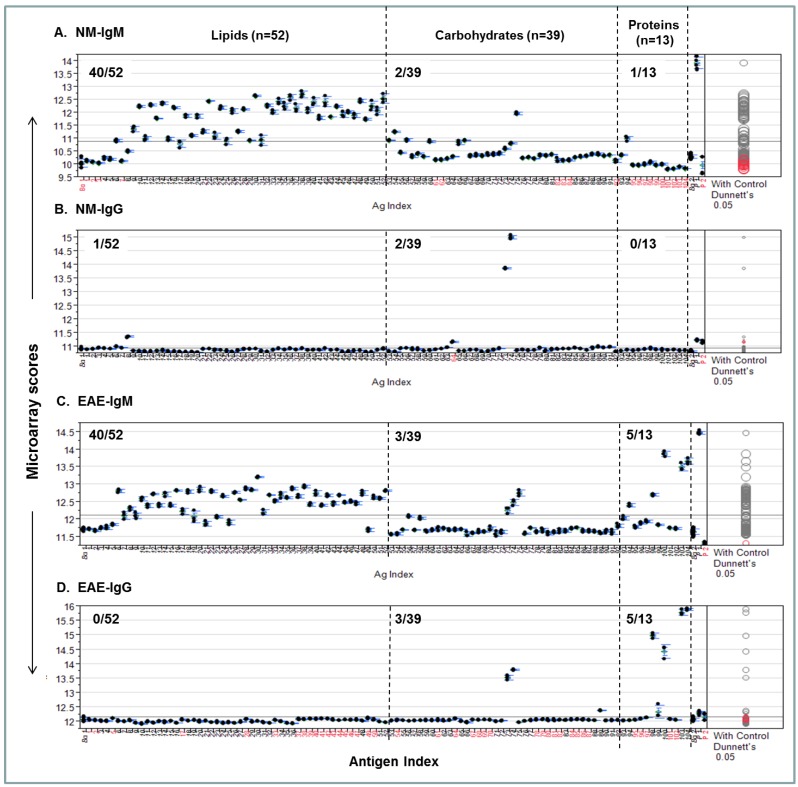
An integrated lipid, carbohydrate, and protein microarray reveals personalized antibody profiles in an EAE model and an age-matched SJL/J normal control (NM). (**A**) NM-IgM antibody profile; (**B**) NM-IgG profile; (**C**) EAE-IgM antibody profile; (**D**) EAE-IgG profile. The two microarray background controls that flank the antigen list are the saline-printed spots (Bg. 1; *n* = 12) and the “virtual spots” in the same array that were captured during microarray scanning (Bg. 2; *n* = 63). Both Bg. 1 and Bg. 2 reflect the sum of substrate background and 1% BSA blocking buffer background. The two printing controls include a fluorescent positive DyeMix (P1) and a visible dye Bismarck (P2), which were spotted for monitoring the process of microarray printing and scanning. The color of the comparison circles and the color of the antigen name listed in *x*-axis are identified in black to indicate that the means of detection is significantly different from those of the means of Bg. 1 and red to refer to non-significant detection as compared to the background. All antigen preparations were indexed with names and descriptions listed in Supplementary Table S1.

A procedure for quantifying personalized antibody profiles using integrated multi-component microarrays is illustrated in Figure 4. Specifically, the microarray datasets for Figure 3 were plotted with microarray scores in the *y*-axis; the antigen items in the *x*-axis; the antigen zones are divided by dashed lines. Dunnett’s test was performed to determine whether the means of a given antigen-specific detection were significantly different from the means of the background values (Bg. 1). Viewing each plot allows us to determine whether the detection is statistically significant and above the mean line of multiplex serum antibody responses in each assay.

Figure 4A shows that 40/52, 2/39, and 1/13 NM-IgM detections were above the mean response line in the lipid, carbohydrate, and protein zones, respectively. By contrast, only 1/52, 2/39, and 0/13 positives in corresponding antigen zones were above the line in the Figure 4B NM-IgG panel. Figure 4C shows the EAE-IgM profile, which has 40/52, 3/39, and 5/13 strong positives in the corresponding zones; the Figure 4D-EAE IgG plot shows 0/52, 3/39, and 5/13 positives above the global means in the corresponding zones.

In summary, this analysis quantitatively illustrates each antibody profile and statistically weights the level of significance for a given detection. Importantly, it provides quantitative comparison of antibody activities among the three major components of antigens, *i.e.*, lipids, carbohydrates, and proteins. By inspecting these plots, the personalized characteristics of the EAE model and an age-matched SJL/J normal control are revealed. The EAE-antibody profile is strikingly featured by markedly increased IgM and IgG responses to a number of myelin proteins and autoantibodies targeting lipids and carbohydrates are also detected.

## 4. Conclusions

In this study, a liposome-based procedure was explored to construct lipid microarrays. Practically, each aqueous suspension of lipid vesicles was printed on nitrocellulose-coated micro-glass slides. With this procedure, the substances that can form liposomes or can be incorporated into liposomes are suitable for microarray construction using existing microarray spotting devices. The liposome arrays constructed by this procedure have achieved the sensitivity required to detect anti-lipid antibodies in blood circulation.

The integrated lipid, carbohydrate, and protein microarrays produced using this approach may have unique value in immunological studies. As illustrated, a serological analysis of the EAE model using such integrated antigen microarrays effectively reveals that the PLP-induced EAE model is characterized by a predominant anti-myelin protein IgG antibody response accompanying IgM responses to a spectrum of autoantigens, including proteins, lipids, and carbohydrates. It appears to be practical to monitor personalized antibody profiles using this technology. Consequently, this biochip platform may have potential use in the practices of personalized healthcare in the future.

Further study is required to examine the long-term stability of liposome arrays and correlate of results between this multiplex liposome array and other individual lipid assays. In considering the structural diversity of lipids and glycolipids, optimized conditions may be required for producing stable *hetero*-liposomes for some targets. It is also important to determine whether chemical modifications of the spotted lipids take place on-chip over time. For example, oxidization may occur under an air-dried condition, which may introduce unexpected neo-epitopes to complicate interpretation of an antibody profiling result. In this regard, optimizing the procedure for a long-term storage of printed liposome arrays is likely a near-term requirement in the development of lipid/liposome array technology.

## Supplementary Files

Supplementary File 1
